# *Penicillium italicum*: An Underexplored Postharvest Pathogen

**DOI:** 10.3389/fmicb.2020.606852

**Published:** 2020-12-04

**Authors:** Aline Midori Kanashiro, Daniel Yuri Akiyama, Katia Cristina Kupper, Taícia Pacheco Fill

**Affiliations:** ^1^Institute of Chemistry, Universidade Estadual de Campinas, Campinas, Brazil; ^2^Advanced Citrus Research Center, Sylvio Moreira/Campinas Agronomic Institute, São Paulo, Brazil

**Keywords:** *Penicillium italicum*, virulence factors, natural products, pathogen host interaction, blue mold disease, blue mold

## Abstract

In the agricultural sector, citrus is one of the most important fruit genus in the world. In this scenario, Brazil is the largest producer of oranges; 34% of the global production, and exporter of concentrated orange juice; 76% of the juice consumed in the planet, summing up US$ 6.5 billion to Brazilian GDP. However, the orange production has been considerable decreasing due to unfavorable weather conditions in recent years and the increasing number of pathogen infections. One of the main citrus post-harvest phytopathogen is *Penicillium italicum*, responsible for the blue mold disease, which is currently controlled by pesticides, such as Imazalil, Pyrimethanil, Fludioxonil, and Tiabendazole, which are toxic chemicals harmful to the environment and also to human health. In addition, *P. italicum* has developed considerable resistance to these chemicals as a result of widespread applications. To address this growing problem, the search for new control methods of citrus post-harvest phytopathogens is being extensively explored, resulting in promising new approaches such as biocontrol methods as “killer” yeasts, application of essential oils, and antimicrobial volatile substances. The alternative methodologies to control *P. italicum* are reviewed here, as well as the fungal virulence factors and infection strategies. Therefore, this review will focus on a general overview of recent research carried out regarding the phytopathological interaction of *P. italicum* and its citrus host.

## Introduction

Citrus is one of the most produced and exported fruit genus in the world (Liu et al., 2012; Papoutsis et al., 2019) being consumed *in natura* or as derived products. Many substances that constitute citrus fruits are essential for humans, being used in medicine and other sectors (Talibi et al., 2014; Al-snafi, 2016), such as flavonoids that have anti-cancer and anti-inflammatory properties (Benavente-García and Castillo, 2008). Brazil is the largest citrus producer and exporter in the world (Lopes et al., 2011; de Vilhena Araújo et al., 2019), mainly of oranges. The country produces 34% of the global orange production and 76% of the juice consumed in the world, generating about 200 thousand direct and indirect jobs, and a contribution to the Brazilian GDP of US$ 6.5 billion (Neves and Trombim, 2017; Bazioli et al., 2019).

**Figure 1:**
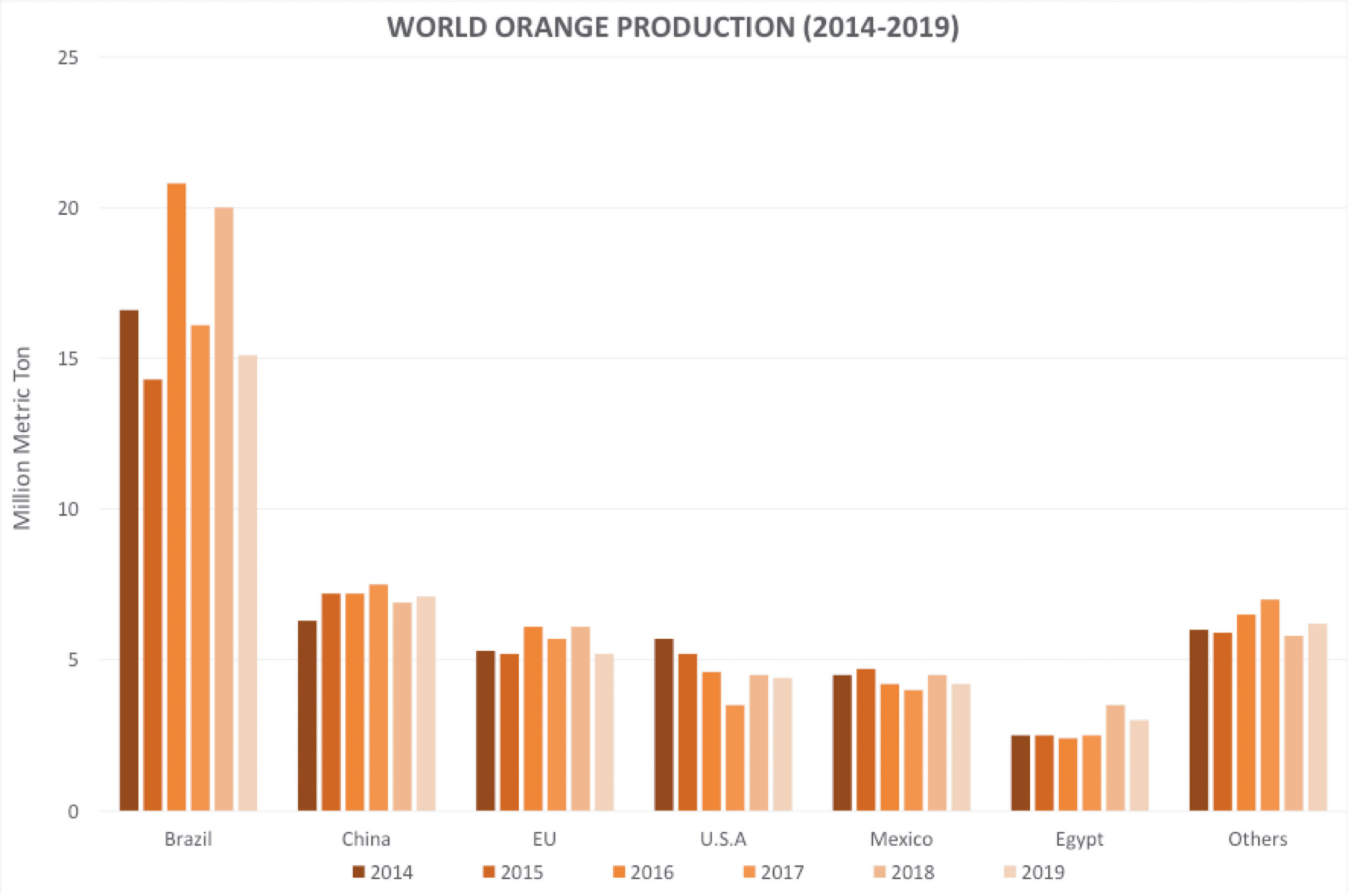
Global orange production in the last 6 years. Source: (USDA, 2020). Accessed on: Jan. 27, 2020.

However, unfavorable climatic conditions in recent years (USDA, 2020), such as warm temperatures and below-average rainfall after the first two blooms and fruit set, and the increasing number of pathogen infections have triggered a considerable decrease in the orange production (Figure 1) (Palou et al., 2002; Tayel et al., 2015; Cunha et al., 2018; Yang et al., 2020). The resulting economic losses are estimated to account for up to 30 to 50% of all production (Singh et al., 2012; Vitoratos et al., 2013; Yun et al., 2013; Aloui et al., 2015; Wan et al., 2017; Youssef and Hussien, 2020).

Due to oranges acidic pH, around 4–5 in healthy fruits (Costa et al., 2019a), most of the orange rot is caused by fungi and not bacteria (Talibi et al., 2014). Phytopathogenic fungi can produce and proliferate mycotoxins, secondary metabolites of low molecular mass produced by filamentous fungi (Amadi and Adeniyi, 2009; Zain, 2011), which are often toxic to the host and other organisms that cohabit the same microenvironment (Zain, 2011; Dukare et al., 2019). Currently, more than 500 different mycotoxins have already been reported, including economically, and toxicologically relevant compounds that threat human and animal life such as: aflatoxins, trichothecenes, fumonisins, zearalenone, ochratoxin, and patulin (Bennett and Klich, 2003; CAST, 2003; Köppen et al., 2010; Medeiros et al., 2012). The FDA has estimated that more than half billion dollars have been invested in mitigating costs due to only three mycotoxins: aflatoxins, fumonisins, and trichothecenes (Bhatnagar et al., 2006).

The most harmful phytopathogenic fungi of oranges are *Penicillium digitatum*, which causes the green mold disease, responsible for about 90% of post-harvest losses (Costa et al., 2019b; Papoutsis et al., 2019), and *Penicillium italicum* Wehmer, the causing agent of the blue mold disease. The latter disease develops more slowly, however, it presents higher resistance to cold (Whiteside et al., 1993; Palou et al., 2002; Iqbal et al., 2012, 2017) and to low water availability (Plaza et al., 2003), easily spreading and contaminating a greater number of healthy oranges. The presence of wounds in the fruit surface is essential for infection by these fungi (Caccioni et al., 1998; Talibi et al., 2014).

Louw and Korsten (2015) noted that *P. italicum* caused significantly large lesions on ambient storage lemon fruit (33.9 ± 11 mm) being also able to cause smaller lesions (19.9 ± 11.0 nm) under cold-storage conditions (86.4 ± 4.5% relative humidity). The lesions growth rates are 4.8 and 1.4 mm/day at ambient and cold conditions storage, respectively. Additionally, infections caused by *P. italicum* showed the first signs of lesion development under cold conditions after 12–13 days, while under ambient conditions, the first signs were observed after 2–3 days (Louw and Korsten, 2015). The symptoms of blue mold disease consist of a watery soaked fruit appearance, soft and discolored (due to the production of pathogenic hydrolytic enzymes such as polygalacturonase and glucosidase (Papoutsis et al., 2019), causing losses of smoothness in infected rind tissue and increasing susceptibility to mechanical damage (Louw and Korsten, 2015). As the white mycelium grows and extend deeper into the infected tissue, it later sporulates into blue conidia (Louw and Korsten, 2015). The infection's proliferation occurs through the spread of fungal spores in the air, being only able to contaminate by direct contact with wounded healthy fruits before or after harvest (Kellerman et al., 2016; Papoutsis et al., 2019). The disease severity increases with fruit maturity. Temperature at the range of 20–25°C and high spore concentration in skin wounds also increases de severity of disease development (Papoutsis et al., 2019).

## Control Methods

Concerning food waste and financial losses, some measures were taken to decrease post-harvest decay due to fungal infections, such as the one caused by *P. italicum*, in which chemical methods are the most used today. Currently, the main pesticides used to control *P. italicum* are sterol demethylase inhibitor (DMI) Zhang et al. (2019) fungicides, like Imazalil (IMZ), Pyrimethanil, Fludioxonil, and Tiabendazole, which are toxic chemicals that are harmful to the fruit and also to human health (Ragsdale and Sisler, 1994; Singh et al., 2012; Papoutsis et al., 2019). Studies demonstrated that *P. italicum* has developed higher resistance to these chemicals as a result of its continued use (Iwao, 1999; Arrebola et al., 2010; Tayel et al., 2015).

### Fungal Resistance

Ghosoph et al. (2007), Hamamoto et al. (2000), and Kiralj and Ferreira (2008) elucidated the mechanism of IMZ resistance in *P. digitatum* as a unique sequence insertion in the PdCYP51 gene promoter region, resulting in an increased production of P450-dependant sterol 14-α-demethylase, affecting IMZ sterol demethylase inhibition capabilities (Erasmus et al., 2015; de Ramón-Carbonell and Sánchez-Torres, 2020).

To further understand the molecular basis of IMZ resistance, Zhang et al. (2020) analyzed the comparative transcriptome profile of two strains of *P. italicum* (Pi-R, highly resistant vs. Pi-S, highly sensitive to DMI fungicides) treated with prochloraz. Several differentially expressed genes were identified in Pi-R, which are probably associated with *P. italicum's* DMI-resistance. Among them, ergosterol biosynthesis-related genes, such as ERG2, ERG11 (CYP51 isoform), and ERG6, which encodes a sterol isomerase, sterol 14-α-demethylase (Martel et al., 2010), and sterol methyltransferase, respectively. Besides those, ATP-binding cassette (ABC) transporter family proteins, multi-drug and toxic compound extrusion (MATE) family proteins and major facilitator superfamily (MFS) proteins have also been up-regulated in fungal resistance. Ergosterol biosynthesis related enzymes have been described as important factors to cycloheximide resistance in *Saccharomyces cerevisiae* (Abe and Hiraki, 2009). Similarly, the ABC transporter family and the MFS proteins were already reported as up-regulated in *P. digitatum* fungal resistance (Nakaune et al., 2002; Sánchez-torres and Tuset, 2011; Wang et al., 2012; Wu et al., 2016b). The similarities between transcriptomic analysis of resistant *P. italicum* and *P. digitatum* strains indicate common resistance factors in both citrus pathogens.

Other metabolism regulating enzymes are thought to play an important role in fungicide resistance. The cascade signaling regulator effect of the mitogen-activated protein kinase (MAPK) regulates a series of other important protein kinases, resulting in stress-induced cell wall remodeling regulation. To further support the importance of MAPK activity in fungicide resistance, Wang et al. (2014) reported that the Hog1-MAPK (PdOs2)-mediated cell wall integrity (CWI) signaling system is involved in *P. digitatum*'s resistance to fludioxonil and iprodione. Since Ca^2+^ and Ca^2+^/calmodulin-dependent kinase (CaMK) are usually linked with MAPK pathway regulation, the overexpression of the CaMK2 gene was evaluated in *S. cerevisiae*, indicating facilitated resistance to azole-fungicides as well as fungal cell wall protective activity against oxidative and heat stresses (Dudgeon et al., 2008; Kumar and Tamuli, 2014). The MAPK/calcium signaling-related genes were once again up-regulated in prochloraz-treated Pi-R strains of *P. italicum*, indicating another important resistance factor in this species. Furthermore, Nicolopoulou-Stamati et al. (2016) confirmed the toxicity to human health of these fungicides, thus being necessary to develop other methods to control post-harvest fungi diseases.

**Figure 2:**
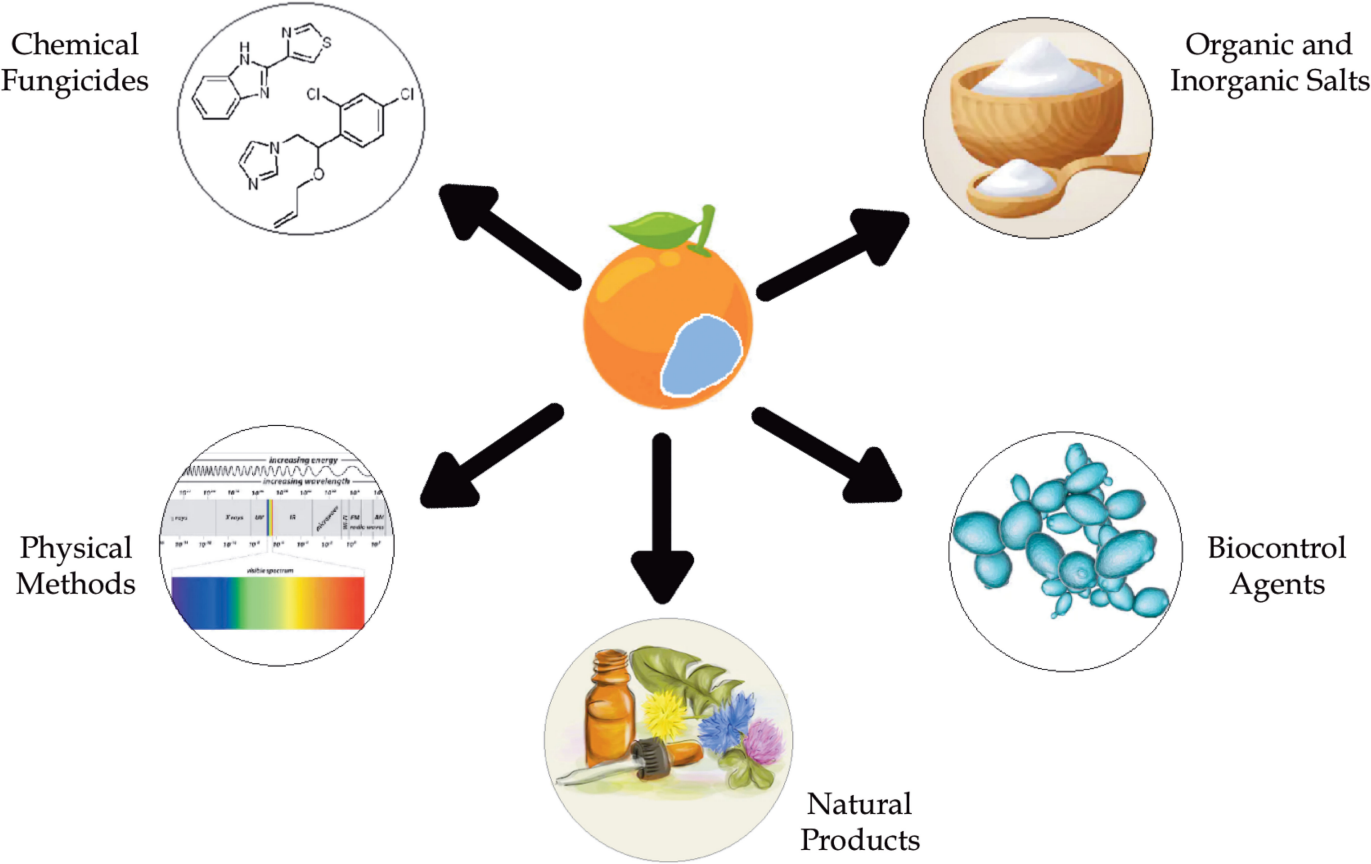
Type of control methods against *P. italicum*.

To address this growing problem, the search for new methods of post-harvest phytopathogen control is being explored. Alternative methods for controlling the blue mold disease include the application of natural products (NPs) found in microbial or plant extracts and essential oils from plants (Table 1) (Solgi and Ghorbanpour, 2014; Trabelsi et al., 2016), the use of organic and inorganic salts (Youssef and Hussien, 2020), biocontrol methods, such as yeasts (Parafati et al., 2016; Cunha et al., 2018; Bazioli et al., 2019), and physical methods (Figure 2).

**Table 1:** Alternative methods based in natural products (NPs) found in microbial or plant extracts and essential oils from plants.

**Organism**	**Natural products**	**Minimum inhibitory concentration (MIC)**	**References**
*Aspergillus, Penicillium*, or *Acetobacter* species	Kojic Acid (KA) combinated with H_2_O_2_	12.8 and 1.5 mm (KA and H_2_O_2_, respectively)	Kim and Chan, 2014
*Aspergillus terreus* SCSIO 41202	Sinulolide I, (9Z, 12Z)-N-(2-hydroxyethyl)-octadeca-9,12-dienamide, dodecanoic acid and decanoic acid	0.031-0.125 mg mL^−1^	Yang et al., 2020
*Bacillus pumilus* (*B. pumilus*)	Volatile Organic Compounds (VOCs): methyl isobutyl ketone, ethanol, 5-methyl-2-heptanone, and S-(-)-2-methylbutylamine	-	Morita et al., 2019
Chinese propolis	Pinocembrin	400 mg L^−1^	Peng et al., 2012
Chinese propolis	Pinocembroside	200 mg L^−1^	Chen et al., 2019c
*Citrus aurantium*	α-terpineol, terpinen-4-ol, linalool, and limonene	2.5 μL mL^−1^	Trabelsi et al., 2016
Cinnamon bark (*Cinnamomum cassia L*.)	Cinnamaldehyde and eugenol	130–398.11 μL mL^−1^	Kanan and Al-Najar, 2009
Citrus fruits	Citral	0.5 μL mL^−1^	Droby et al., 2008; Tao et al., 2014b
Citrus fruits	Octanal	1.0 μL mL^−1^	Klieber et al., 2002; Droby et al., 2008; Tao et al., 2014a
Garlic oil nanoemulsion (GO NE)	Dimethyl trisulfide, diallyl disulfide, diallyl sulfide, diallyl tetrasulfide, 3-vinyl-4H-1,2-dithiin, diallyl trisulfide, 1,4-dimethyl tetrasulfide, methyl allyl disulfide and methyl allyl trisulfide	0.01265%	Ding et al., 2014; Li et al., 2014; Li W.-R. et al., 2015; Long et al., 2020
*Laminaceae spp*.	Carvacrol and thymol	-	Pérez-Afonso et al., 2012
*Peganum harmala L. (harmal seeds)*	Harmine, harmaline, and tetrahydroharmine (THH) alkaloids	263.03–514.81 μL mL^−1^	Kanan and Al-Najar, 2009
Pomegranate (*Punica granatum*) peel extract (PGE)	Phenolic compounds	-	Nicosia et al., 2016; Pangallo et al., 2017
*Populus euramericana* cv. “Neva” (poplar buds)	Flavonoids of pinocembrin, chrysin, and galangin	-	Yang et al., 2016
*Ramulus cinnamomi*	Cinnamic acid and cinnamaldehyde	-	Wan et al., 2017
*Sedum aizoon L*. (FSAL)	Gallic acid, quercetin and kaempferol	1.75 mg mL^−1^	Luo et al., 2020
*Streptomyces globisporus* JK-1	Dimethyldisulfide, dimethyltrisulfide and acetophenone	-	Li et al., 2010
*Thymus* species (*T. leptobotris, T. riatarum, T. broussonnetii subsp. hannonis, and T. satureioides subsp. pseudomastichina*)	Thymol, carvacrol, geraniol, eugenol, octanal, and citral	<500 μL L^−1^	Boubaker et al., 2016
*Thymus leptobotris*	Thymol and carvacrol	-	Ameziane et al., 2007
*Thymus vulgaris L*.	Thymol	0.13 e 0.50 μL mL^−1^ (mycelium growth and spore gemination, respectively)	Vitoratos et al., 2013

### Natural Product and Plant Extract Applications

Several studies indicate that secondary phenolic metabolites, flavonoids, anthraquinones, acetaldehyde, alkaloids, allicin, benzaldehyde, benzyl alcohol, (E)-2-hexanal, ethanol, ethyl benzoate, ethyl formate, glucosinolates, hexanal, isothiocyanates, isoverbascocide, lipoxygenases, methyl salicylate, phenylpropanoids, quinones, saponins, sterols, tannis, terpenes, verbacocide play a considerable role in *P. italicum* fungal control (Palou et al., 2002; Askarne et al., 2012; Papoutsis et al., 2019). The isoprenoid citral, naturally produced by citrus fruits with two isomers, has been reported for its antifungal activity against *P. digitatum, P. italicum*, and *G. citri-aurantii*. Citral solutions and vapors in different concentrations have the ability to inhibit fungal growth, spore germination, and germ tube growth of these pathogens (Klieber et al., 2002; Droby et al., 2008).

Chen et al. (2019b) explored the antifungal capacity of the flavonoid pinocembroside, which is the main antifungal component present in the Chinese propolis, through optical and scanning electron microscopy (SEM) analysis to evaluate changes in hyphal morphology. Due to the low toxicity described, in addition to the higher antioxidant potential, and strong antibacterial properties, the compound could be applied as a promising method for the control of the blue mold disease. Studies have shown that the complete inhibition of *P. italicum's* mycelial growth by pinocembroside occurred at a minimum inhibitory concentration (MIC) of 200 mg/L, half the concentration required for the effect of both pinocembrin (Peng et al., 2012), and sodium dehydroacetate. Higher concentrations of the compounds 7-demethoxytylophorine (1.56 μg/mL) (Chen et al., 2019b), citral (0.5 μL/mL) (Tao et al., 2014b), and octanal (1.0 μL/mL) (Tao et al., 2014a) were required for activity. Despite the known mechanism of action of flavonoids, which inhibit the mycelial growth mainly by causing cell membrane damage accompanied by the outflowing of certain intracellular components interrupting metabolic respiration and disturbing enzymes related to fungus energy production, the antifungal mechanisms of pinocembroside have not been fully elucidated. However, the authors suggest that the mechanism is related to changes in the cell membrane structure, increasing permeability, accelerating lipid peroxidation and reducing the activity of antioxidant enzymes (Chen et al., 2019b,c).

Some bioactive compounds derived from fatty acids can also be effective in fungal growth control as they move easily into fungal cells to exert their toxic effects. In the study of the ethyl acetate extract from the marine-derived fungus *Aspergillus terreus* SCSIO 41202, Yang et al. (2020) found four bioactive compounds derived from fatty acids that had antifungal activity: sinulolide I, (9Z, 12Z)-N-(2-hydroxyethyl)-octadeca-9,12-dienamide, dodecanoic acid, and decanoic acid. The antifungal activity was related to the long aliphatic chain and the acidic group of these compounds, presenting MIC values between 0.031 and 0.125 mg/mL, indicating that these compounds may be an effective alternative method for the control of *P. italicum* (Yang et al., 2020).

In addition, there are some volatile NPs produced by the fruit that collaborates with the fungus growth. According to Droby et al. (2008), the meroterpenes limonene, α-pinene, β-pinene and myrcene were stimulatory for *P. digitatum* and *P. italicum*, probably serving as host recognition signaling, since they also presented small inhibitory effect to non-citrus pathogens. Although these are promising results, it is necessary more studies concerning the basic biochemistry and molecular understanding of how these chemicals trigger germination or inhibition activities in *P. italicum* and *P. digitatum*. New studies on this phytopathological relationship are increasingly needed, since understanding the fungus-host interaction is an essential step in the development of new, safe, and efficient control methods.

In the general review of non-chemical methods used for prevention of postharvest fungal rotting caused by *P. digitatum* and *P. italicum*, Papoutsis et al. (2019) discovered that, in general, extracts from isolated or combined plants are promising fungicides with well-documented antifungal activity, low phytotoxicity, systemic mechanism of action, decomposability, and low environmental toxicity. Methanolic extracts from cinnamon bark (*Cinnamomum cassia L*.) and sticky fleabane leaves (*Inula viscosa* L.) have been associated with a high amount of phenols, flavonoids, and anthraquinones, while the antifungal activity of harmal seeds (*Peganum harmala* L.) has been attributed to its high concentration of phenolic compounds and alkaloids (Kanan and Al-Najar, 2009). Phenolic extracts from pomegranate (*Punica granatum*) peels didn't cause any phytotoxic syndrome in citrus, being an excellent candidate for fungal control (Nicosia et al., 2016; Pangallo et al., 2017).

In general, the molecular mechanisms involved in growth inhibition by plant extracts are: the inhibition of DNA gyrase, responsible for DNA biosynthesis, energy production metabolism and cellular respiration; inactivation of essential enzymes and the function of genetic material; and interference with membrane permeability, modification of the fungal cell structure., (Telezhemetskaya and D'yakonov, 1991; Cushnie and Lamb, 2005; Xu et al., 2011; Wu et al., 2013; Silva et al., 2014; Yang et al., 2016; Pangallo et al., 2017). In addition, plant extracts can stimulate the host's defensive responses, initiate oxidative stress and react with the pathogen's cell membrane proteins (Yang et al., 2016; Papoutsis et al., 2019).

The flavonoids from *Sedum aizoon* L. (FSAL), which are mainly composed of gallic acid, also contain significant amounts of quercetin and kaempferol, presenting antifungal activity against plant pathogens. Transcriptomic analyses performed by Luo et al. (2020) on *P. italicum* grown in the presence of FSAL indicated the flavonoids inhibitory activity against *P. italicum*. Eighty-three genes encoding plasma membrane and 23 genes related to the hyphal cell wall were differentially expressed in this study, resulting in cell wall disintegration and hyphal collapse. Furthermore, many critical oxidative stress resistance-encoding genes were up-regulated in the presence of FSAL, indicating an increase in reactive oxygen species (ROS) levels.

Another promising category of antifungal agents is antimicrobial peptides (AMPs), which are polypeptides synthesized by ribosomes and encoded by genes that are present in a range of organisms. Most AMPs are cationic, amphipathic and have low phytotoxicity (Wang et al., 2018). Wang et al. (2018) found that AMPs could reduce the growth of *P. italicum in vitro* by 70% or more and inhibit the progression of the disease *in vivo*, by changing the permeability of the cell membrane and the structure of the cell wall. Inhibition assays were performed utilizing 64 μM of PAF56 (amino acid sequence: GHRKKWFW), and the molecular inhibition mechanism was confirmed. The peptide acts on fungal membranes and cell wall, resulting in fungal death. Essential Oils (EOs) are also showing excellent results against phytopathogens, as they are natural products safe to human health and to the ecosystem, in addition to producing low levels of traceable residues and having a smaller chance of inducing pathogen resistance since they contain several volatile substances, each with a different mechanism of action (Ameziane et al., 2007; de Morais, 2009; Wu et al., 2016a; Papoutsis et al., 2019). Research using essential oils such as those obtained from *Cinnamomum zeylanicum, Citrus aurantium, Thymus vulgaris, Thymus leptobotris, Thymus riatarum, Thymus broussonnettii, Eugenia caryophyllata Thunb*, Bergamot, Thyme, Tea tree, and *Thymus capitatos* may be promising to inhibit *P. italicum* (Palou, 2014; Boubaker et al., 2016; Chen et al., 2019a; Dukare et al., 2019; Papoutsis et al., 2019). Also, thymol, carvacrol and the mixture of both (Pérez-Afonso et al., 2012) proved to be effective in controlling this fungus. In their experiments with oranges, Li et al. (2010) found that the volatile organic compounds (VOCs) produced by *Streptomyces globisporus* JK-1 dimethyl trisulfide and acetophenone showed effective inhibitory activity *in vitro* and *in vivo*, being the first study to confirm such antifungal action. VOCs produced by bacterial strains have been reported to inhibit fungal mycelial growth and, in some cases, to be responsible for the induction of plant resistance in stressed hosts (Girón-Calva et al., 2012; Martins et al., 2019).

Other studies confirmed the antifungal effects of Garlic Oil (GO) (Li et al., 2014; Long et al., 2020), which is rich in organosulfur compounds, mono to hexa diallyl sulfides and vinyl dithiin isomers, such as dimethyl trisulfide, diallyl disulfide, diallyl sulfide, diallyl tetrasulfide, 3-vinyl-4H-1,2-dithiin, diallyl trisulfide, 1,4-dimethyl tetrasulfide, methyl allyl disulfide, and methyl allyl trisulfide. However, the low stability, high volatility, and hydrophobic properties limit GO applications for antifungal studies, in addition to having antioxidant activity, which aids the development of fungal infection. To work around these problems, Long et al. (2020) formulated a GO nanoemulsion (NE) through ultrasonic technique. This method allows increased bioavailability of GO as well as reducing the cost of sterilization. The NE was composed of the surfactants Span 80 and Tween 80 (Smix), which do not show antifungal activity in the absence of GO. Research involving GO NE as an antifungal controller managed to reveal its antifungal mechanism on *P. italicum* based on structural and molecular analyses, using micro-confocal and surface-enhanced Raman spectroscopy that showed the inhibition of mycelia in the medium and the destruction of membranes and the cell wall of the fungus. On the other hand, the authors are still looking for alternatives to remove the strong odor of garlic to make it applicable on a commercial scale.

Another possibility for controlling the blue mold disease is the use of chemosensitizing antifungal agents combined with oxidative stressors. In the studies conducted by Kim and Chan (2014), the antifungal efficacy of Kojic Acid (KA) produced by certain filamentous fungi of the *Aspergillus* and *Penicillium* genera (Liu et al., 2014) was analyzed considering different treatment temperatures in the presence or absence of H_2_O_2_. In treatments at relatively high temperatures (35–45°C) H_2_O_2_ was more efficient, however, at higher temperatures (55°C) this efficiency decreases. KA, on the other hand, does not change its effectiveness with increasing temperature. As KA induces the generation of reactive oxygen species in cells, such as in macrophages, stimulating phagocytosis, the combined chemosensitization (KA + H_2_O_2_) generated high oxidative stress, possibly being the mechanism of increased activity resulting from this combination. Studies have shown that H_2_O_2_ damages the cellular integrity of *P. italicum* strains, inhibiting the cell division cycle, in the antioxidant defense and metabolism of the fungus. In short, the combined treatment of KA and H_2_O_2_ can be promising in the control of fungal pathogens, since KA is a safe natural compound, as demonstrated by Fickova et al. (2008) through cytotoxic assays, being used in cosmetic products and medicines (Niwa and Akamatsu, 1991; Fickova et al., 2008; Rodrigues et al., 2011; Kim and Chan, 2014; Liu et al., 2014; Saeedi et al., 2019). Moreover, the treatment can be done in considerably low temperatures, which reduces damage to crops, the environment and health. However, it should be considered the lack of sensitivity of several strains of *P. italicum* to this treatment, explained by the fact that KA is produced by different strains of *Penicillium*.

The study of Morita et al. (2019) explored bacteria that produce volatile organic compounds as biocontrol agents. The TM-R strain of the gram-positive bacterium *Bacillus pumilus* (*B. pumilus*) showed the greatest antifungal activity among 136 bacterial isolates. Small and large-scale tests were performed on four types of agar (Nutrient Agar—NA, Tripto-Soya Agar—TSA, Luria-Bertani Agar—LBA, and TM Enterprise Agar—TMEA). Despite the limitations to identify VOCs due to their low concentrations, complexity of their compositions and differences of the culture medium, four predominant VOCs were detected and correlated to the antifungal activity, namely: methyl isobutyl ketone, ethanol, 5-methyl-2-heptanone, and S-(-)-2-methylbutylamine. Regarding the antifungal effect, *B. pumilus* TM-R was able to inhibit from 95 to 100% (depending on the medium) of the growth of *P. italicum* in the plaque test and 93% in the large-scale test in TMEA. Since it does not produce hemolysin or DNase, *B. pumilus* TM-R has a high chance of not being pathogenic to humans, which makes it very promising in commercial applications. In addition, this study proved that the bacteria promoted the growth of one of the tested fungus species (*Aspergillus niger*), which obtained growth values of 36% in the 12 L TMEA test and 9% in the plaque test, which can be a potential problem in antifungal treatment if it stimulates the growth of another citrus pathogen not studied.

### Organic and Inorganic Salts

In the United States, the use of sodium carbonates and bicarbonates is allowed to control mold, however, the disposal of the used substances proved to be a problem due to their high pH, sodium content and conductivity of sodium carbonate (Smilanick et al., 2008; Li et al., 2010). Potassium sorbate, ammonium bicarbonate, calcium polysulfide, sodium ethyl paraben and sodium hydrosulfide also had their antifungal activities tested (Papoutsis et al., 2019). In fact, the mechanism of action of these salts has not been determined yet, which have greater efficacy when associated with other methods. However, the osmotic stress generated by high concentrations of salts applied to the fruit can decrease fungal population, in addition to influencing the growth of the pathogen as fungi thrive best at acidic and neutral pH. These salts combined with waxes or other antifungal methods can induce an increase in SOD (superoxide dismutases) (Parafati et al., 2016), PAL (phenylalanine ammonia lyase) (Chen et al., 2019a), CHI (chitinase) (Pangallo et al., 2017; Chen et al., 2019a), ROS (reactive oxygen species) (Pangallo et al., 2017), and β-1,3-glucanases levels (Santos et al., 1978, 1979).

### Biocontrol Agents

Recent studies increasingly indicate that the products generated by biocontrollers are promising agents in the antifungal activity, being safe for the environment and to human health (Table 2). Killer yeasts can secrete lethal protein toxins or low molecular weight glycoproteins to other susceptible yeasts (Aloui et al., 2015), fungi and filamentous bacteria (Pimenta et al., 2008). The advantages of using killer yeasts as biocontrol agents are based on their adaptive characteristics, the low cost to quickly produce large amounts of yeast, the absence of the production of toxic compounds and the ability to colonize and survive on the fruit surface for a long period of time and in various environmental conditions. These advantages make killer yeasts possibly better antagonists than other sources, acting by adhering to the specific site, such as other yeasts or pathogenic cells, and forming colonies in the wound that compete with the fungus for nutrients. They secret specific enzymes and antimicrobial substances, which can be soluble or volatile, and so they help to inhibit the pathogen, by forming a biofilm on the inner surface of the wounds, which act as a protective layer so that the fungus cannot progress the infection process (Liu et al., 2019). The efficiency of the yeast *Saccharomycopsis schoenii* (*S. schoenii*) against three phytopathogenic species of *Penicillium* was evaluated, with a 35.7% reduction in the severity of the disease in oranges. Despite some desirable effects of *S. schoenii* as a fungal controller, a high concentration of this yeast was necessary for its effectiveness, as also demonstrated in other yeast species (Pimenta et al., 2008). According to Ferraz et al. (2016), the yeast *Candida azyma* presented potential as a biological control agent against *G. citri-aurantii* and ability to produce killer toxin, as a mechanism of action. To Cunha et al. (2018), the killer activity from *C. stellimalicola* strains might be the main action mechanism involved in *P. italicum* biocontrol, when these yeasts were preventively applied on citrus fruits for the blue mold control.

**Table 2:** Biocontrol methods against *P. italicum*.

**Antagonist agent**	**Mechanism of action**	**References**
*Candida oleophila*	Increase phenylalanine ammonia lyase activity and accumulation of the phytoalexins such as umbelliferone, scoparone, and scopoletin, which led to resistance induction.	Droby et al., 2002; Liu et al., 2019
*Candida stellimalicola*	“Killer” activity, inhibition of conidial germination, and stimulates production of chitinase.	Cunha et al., 2018
*Cryptococcus laurentii* associated with cinnamic acid	Loss of membrane integrity which led to leakage of cytoplasmic materials and death of the fungal pathogen	Li J. et al., 2019
*Debaryomyces hansenii*	Competition for space and nutrients	Droby et al., 1989; Chalutz and Wilson, 1990; Hernández-Montiel et al., 2010
*Kazachstania exígua* and *Pichia fermentans*	“Killer” activity	Comitini et al., 2009
*Metschnikowia citriensis*	Iron depletion, biofilm formation, and adhesion to mycelia.	Liu et al., 2019
*Metschnikowia pulcherrima*, and *Aureobasidium pullulans*	Competition for nutrients and influence in superoxide dismutase and peroxidase activities, which led to fruit resistance induction.	Parafati et al., 2016
*Pseudozyma antarctica*	Direct parasitism, which causes fungal cell wall degradation.	Liu et al., 2019
*Saccharomyces cerevisiae*	“Killer” activity and competition for space and nutrients	Comitini et al., 2009; Platania et al., 2012; Kupper et al., 2013; Cunha et al., 2018
*Saccharomycopsis schoenii*	Predation, competition for nutrients and other antagonistic interactions	Pimenta et al., 2008
*Wickerhamomyces anomalus* (or *Pichia anomala*)	“Killer” activity based on β-glucanase production, competition for nutrients, fruit resistance induction, and antibiosis.	Comitini et al., 2009; Platania et al., 2012; Aloui et al., 2015; Parafati et al., 2016

In another analysis with biocontrol agents, Perez et al. (2016) isolated 437 strains of native yeasts from leaves and fruits of citrus plants as well as water from washing lemon peels, in order to investigate the Killer potential against citrus pathogens. The study identified, through the analysis of the D1/D2 sequence of the 26S rDNA gene, six different genera: *Pichia* (8.1%), *Saccharomyces* (13.5%), *Kazakhstan* (40.5%), *Wickerhamomyces* (2.7%), *Clavispora* (8.1%), and *Candida* (21, 7%). Three types of analysis were performed for determining how many strains had the killer phenotype: eclipse assay (22 strains−5%), diffusion plate technique (30 strains–6.9%), and diffusion plate with addition of NaCl 2% (37 strains−8.5%). As the pH proved to be of vital importance for the killer activity, these analyses were carried out at a pH equal to 4.5, which showed to be another advantage, since the pH of the infected fruit becomes more acidic (around 5). Regarding the antifungal effect, 11 strains of *P. italicum* had growth inhibition of ≥40%; 18 strains were inhibited between 16 and 39%; and the remaining 8 strains showed ≤15% inhibition. *S. cerevisiae* (137) and *Kazachstania exigua* (120) strains showed protective properties against the attack of *P. italicum*. The authors also managed to define that the killer and resistance genes were located on the same plasmid (~4 kb in size) and concluded that despite the positive results, more studies should be performed since species such as *Candida catenulata, Candida Pararugosa*, and *Clavispora lusitaniae* are related to infections in immunocompromised patients.

### Physical Applications

In addition to these methods involving plant extracts, salts and biocontrol agents, new alternatives involving physical methods are being analyzed and tested for the control of post-harvest pathogens. One involves ionizing irradiation, such as gamma and x-ray, and non-ionizing irradiation, such as UV and Blue Light irradiation (Papoutsis et al., 2019). The fruit is exposed to a certain distance from a lamp that radiates UV for a certain period, with varying intensity of treatment. Many studies have focused on the treatment with UV-B and UV-C, however, at high intensities, the latter can alter the fruit's flavor and affect its quality. UV-B presents less harmful effects compared to UV-C, which reduces the incidence of blue mold. The mechanisms of action of UV irradiation are divided between direct, due to the absorption of radiation by the surface of the fungus that inactivates its conidia, and indirect, by the induction of metabolic and anatomical alterations in the citrus flavedo, increasing the resistance of the fruit against the pathogen, making their cell walls thicker (Yamaga et al., 2016; Ruiz et al., 2017; Papoutsis et al., 2019). In addition, after treatment, there is an accumulation of polyphenols and phytoalexins in the flavedo, which are secondary metabolites with antifungal activities. Although the mechanisms of action of Blue Light (which radiates between 400 and 500 nm) have not been clarified yet, some authors suggest that there are direct and indirect actions such as UV irradiation, which damages the morphology and sporulation of the fungus and regulates the metabolic pathways of plant tissues via up-regulated expression of the phospholipase A2 (PLA2) gene. Concerning ionizing irradiations, gamma irradiation is a promising treatment because it is able to delay the ripening of the fruit, since it inhibits ethylene production, as well as the respiration rate, which regulates the enzymatic activity related to the elimination of free radicals. Thus, the gamma irradiation penetrates the fungus, damaging its physiology. However, this treatment impairs the quality of the fruit and there are not many studies on its action concerning *P. italicum*. In addition, it is a strategy with high public resistance and fear of application, due to the popular negative view on this type of irradiation. In this sense, further analysis is still needed to reduce the treatment's impact in the fruit, in addition to promoting greater acceptance by the population about the potential benefits of gamma irradiation. The ionizing x-ray irradiation promotes water photolysis, generating free hydroxyl and hydrogen radicals, stimulating physiological functions in living organisms (Vaseghi et al., 2018; Papoutsis et al., 2019). Furthermore, x-rays can induce the synthesis of antifungal NP such as phytoalexins, scoparone, and scopoletin, but their effectiveness is dependent on the combination with carbonic acid salts (Palou et al., 2007; Rojas-argudo et al., 2012; Papoutsis et al., 2019). Like gamma irradiation, the x-ray is not a well-regarded method and will possibly work better in association with other methods, which still need to be explored (Papoutsis et al., 2019). Finally, the last physical method discussed by Papoutsis et al. (2019) is the treatment with hot water, which is already used to reduce the deterioration caused by pathogens and to increase the useful life of the fruit. The fruits can be dipped or sprayed with hot water (52–53°C and 62°C, respectively), which can instigate the accumulation of CHI and β-1,3-glucanases and disinfect the fruit from the spores of *P. italicum*.

In general, these studies established the basis for better understanding some mechanisms of antifungal treatment, as well as several promising possibilities for the control of the blue mold disease (Luo et al., 2020). However, although there are many promising alternatives, there are none that have the same efficiency as the current commercialized chemicals (Palou et al., 2002; Talibi et al., 2014) and there are not enough studies to prove that the exchange of chemical compounds currently used by physical and biological alternatives have satisfactory efficacy, well-documented antifungal activity, low phytotoxicity, and environmental toxicity, economic and process viability (Papoutsis et al., 2019).

Papoutsis et al. (2019) proposes some parameters to be considered in order to develop new antifungals: (a) being effective even after a short treatment period, (b) the quality of the fruit should not be negatively affected, (c) the minimum effective dose must be considerably low, (d) the efficacy of the product cannot be affected by external conditions, (e) low residual activity, in addition to being non-toxic to human health, and (f) not having ample fungal activity against multiple phytopathogens. In addition, the authors also point out that there should be acceptance of the new control methods by consumers so that there are no significant drops in consumption (Talibi et al., 2014).

Furthermore, *P. italicum* is more resistant to the described antifungal compounds, such as essential oils from citrus fruits (Caccioni et al., 1998) and from some plant extracts (Vitoratos et al., 2013), which instigates the need to focus attention on this mold, once that there are few studies aimed at trying to understand the mechanisms of infection of this phytopathogen, the NPs produced *in vivo* during infection and their possible toxicities against the host and human health.

## Virulence Factors

**Figure 3:**
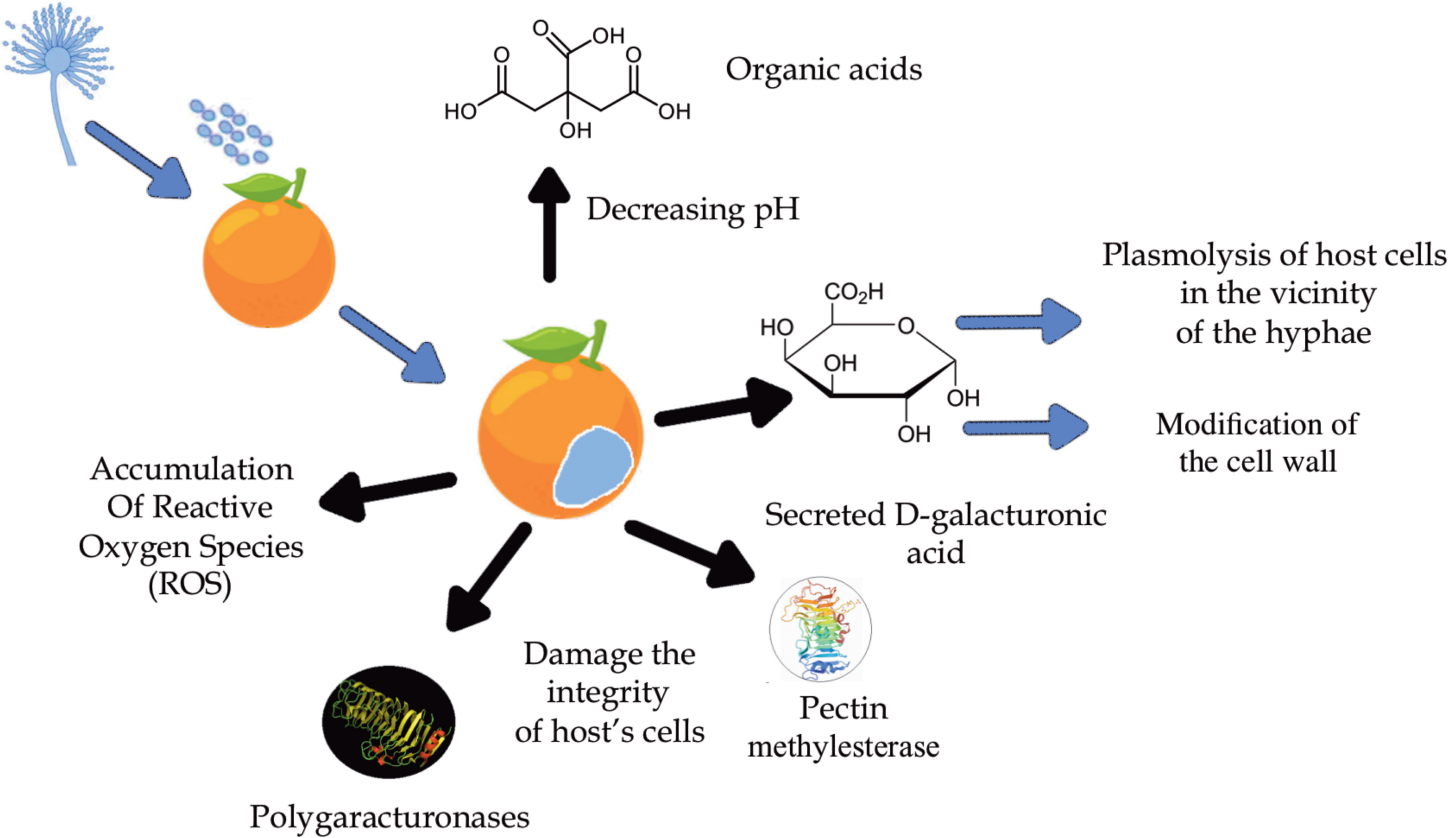
Main virulence mechanisms of host-pathogen interaction.

In order to develop new and safer strategies for effective fungal control against the blue mold disease, it is important to understand the molecular mechanisms of the fungi-host interactions (Figure 3), as well as the pathogenicity and disruption of the fruit's defensive systems (Cheng et al., 2020). Regarding infection in orange, the main virulence and colonization factor known in this phytological interaction are promoted by the hydrolytic enzymes polygalacturonases (PG) produced by *P. italicum* and by other fungi that cause tissue maceration or fruit rot (Prusky et al., 2004; Papoutsis et al., 2019). These enzymes work better at lower pH and, since *P. italicum* is able to acidify the environment with the accumulation of organic acids, especially citric acid, the activity of these enzymes is favored during infection (Prusky et al., 2004). This knowledge suggests that pH is a regulator of gene expression, as it ensures that genes encoding extracellular enzymes are expressed, such as the PEPG1 of the polygalacturonase (PG) enzyme (Prusky et al., 2004). In addition to PG, the enzymes pectate lyase (PL) and pectin lyase (PNL) are also responsible for fruit tissue maceration. However, instead of catalyzing hydrolytic cleavage as PG does, PL and PNL act by splitting the α-glycosidic bond between galacturonic acid residues by trans elimination (Alana et al., 1990). Although *P. italicum* produces only one type of PNL, this strategy seems to be more effective since PNL has a greater stability compared to the others, being active in different culture media and a wide temperature and pH range, proving to be important in the process pathology of *P. italicum* (Alana et al., 1990).

Li T. et al. (2019) suggested in their studies that the modification/degradation of the cell wall caused by enzymatic and non-enzymatic factors is a crucial strategy for the infection of the fruit promoted by *P. italicum*. Increased bioactivities of both PG and pectin methylesterase (PME) and the increased expression levels of xyloglucanendotransglucosylases/hydrolases (XTH) help disassemble the cell wall and damage the integrity of the host's cells. The modification of the cell wall polysaccharides was indicated by the decrease in acid-soluble pectin (ASP) and hemicellulose as well as the increase in water-soluble pectin (WSP), all symptoms caused by the infection of *P. italicum*. The accumulation of reactive oxygen species (ROS) is also observed, promoted by the reduction of antioxidant metabolites as well as the activity of antioxidant enzymes such as superoxide dismutase (SOD), catalase (CAT), peroxidase (POD) (Chen et al., 2019a), and ascorbate peroxidase (APX).

Furthermore, histopathological studies in the tissues of citrus peels infected by both *P. italicum* and *P. digitatum* showed extensive demethylation of pectin, edema of the cell wall and plasmolysis of cells in the vicinity of the hyphae. The dissolution of the cell wall did not occur until hyphae penetration. The reported symptoms of the early stages of their development are linked to a high accumulation of D-galacturonic acid secreted by these fungi (Hershenhorn et al., 1990).

Another mechanism of *P. italicum* infection is the downregulation of hydroxyproline-rich glycoprotein (HRGP) and germin-like protein (GLP) gene expressions, which are related to fruit cell wall. At the transcriptional level, *P. italicum* induced the modification of the cell wall by increasing the expression of the XTH21, XTH29, XTH33, and Expansin-A16 genes, which contribute to cell wall degradation. Although the study revealed an increase in lignin production, which is related to a citrus defense mechanism, infection of the fruit by *P. italicum* was still successful (Li T. et al., 2019).

Li T. et al. (2019) and Yin et al. (2020) observed that Dicer-type genes, which encodes an RNase III-like endonuclease, an important component of RNAi metabolism, play an important role in the condition and pathogenicity of *P. italicum. One* of the proposed virulence mechanisms is the cross-kingdom RNA interference (ck-RNAi), which is a natural phenomenon where small interference RNAs (siRNAs) are transferred between host and pathogen. In this case, only a few siRNAs from *P. italicum* manage to cross the borders of the plant and silence its defensive genes, facilitating virulence, while most remaining siRNAs act endogenously.

Genome analyses indicated that nine carbohydrate active enzymes (CAZyme) families related to pectin, namely: PL1, PL3, PL4, GH28, GH78, GH95, GH105, CE8, and CE12 are encoded by *P. italicum* genes. Since cell wall of fruit cells present an abundant amount of pectin, most of the virulence mechanisms are related to the modification/degradation of polysaccharides (Li B. et al., 2015). *P. italicum* also has a secretome of 662 predicted secreted proteins (7.1% of the proteome) and, considering that secretomes contain abundant proteases, they also help the fungus in virulence, since the proteases allow the fungus to exploit various environmental nutrients and neutralize defense responses based on host proteins. Table 3 shows the main virulence factors:

**Table 3:** Main virulence factors of *P. italicum*'s infection.

**Action**	**Agents responsible**	**References**
Accumulation of reactive oxygen species (ROS)	Reduction of antioxidant metabolites and activity of antioxidant enzymes such as SOD, CAT, POD, and APX	Chen et al., 2019a
Damage the integrity of the host's cells	Increase PME, PG, and XTH	Li T. et al., 2019
Decreasing pH	Organic acids, especially citric acid	Prusky et al., 2004
Demethylation of pectin	Accumulation of D-galacturonic and CAZymes such as PL1, PL3, PL4, GH28, GH78, GH95, GH105, CE8 e CE12	Hershenhorn et al., 1990; Li T. et al., 2019; Yin et al., 2020
Fruit tissue maceration	PG, PL and PNL	Alana et al., 1990; Prusky et al., 2004; Papoutsis et al., 2019
Modification of the cell wall	Decreasing ASP and hemicellulose. Increasing PME, PG, XTH, WSP, D-galacturonic acid and expression of the XTH21, XTH29, XTH33, and Expansin-A16 genes	Hershenhorn et al., 1990; Li B. et al., 2015; Li T. et al., 2019
Neutralization or silencing of defense of host	ck-RNAi and proteases contained in secretomes	Li B. et al., 2015; Li T. et al., 2019; Yin et al., 2020
Plasmolysis of cells in the vicinity of the hyphae	Accumulation of D-galacturonic acid	Hershenhorn et al., 1990

*ASP, Acid-soluble pectin; APX, Ascorbate peroxidase; CAT, Catalase; CAZyme, Carbohydrate active enzymes; ck-RNAi, Cross-kingdom RNA interference; PG, Polygalacturonases; PL, Pectate lyase; PNL, Pectin lyase; PME, Pectin methylesterase; POD, Peroxidase; WSP, Water-soluble pectin; XTH, Xyloglucanendotransglucosylases/hydrolases*.

Interactions between pathogen and fruit remain relatively unexplored (Cheng et al., 2020), mainly regarding the lack of clarity of the virulence mechanisms of the phytopathogen *P. italicum*. Furthermore, the development of these analyses is hampered by the difficulty in individually identifying *Penicillium* species, especially those whose genetics are remarkably similar. However, this distinction is essential for estimating fungal resistance and adapting effective strategies to control decomposition and mycotoxin accumulation.

## Secondary Metabolites Produced by *P. italicum*

Only few studies are dedicated to understand the infection mechanisms of *P. italicum* and so far, no secondary metabolites produced by this fungus are described as virulence factors. It is well documented that these molecules enhance disease development, as well as inhibit or hinder fruit's defensive system in different pathogen-host interactions. Thus, comprehending the secondary metabolites produced by the fungus during infection including their role in virulence is crucial when searching for new strategies to inhibit disease spread.

Frisvad and Filtenborg (1983) performed tests with species of *Penicillium* to analyze mycotoxins and other secondary metabolites (Table 4) and detected two NPs produced by *P. italicum*. However, structure elucidation was not possible in this study. Thus, it became unfeasible to characterize them as mycotoxins or not. Nevertheless, further analysis by Frisvad and Samson (2004) did not add *P. italicum* among mycotoxigenic producers but reported secondary metabolites such as deoxybrevianamide E **(1)**, xanthocyllin **(3)**, and PI-3 **(9)** (Arai et al., 1989). Other studies developed by Faid and Tantaoui-Elaraki (1989), Scott et al. (1974), and Arai et al. (1989) and collaborators have also managed to identify some NPs produced by *P. italicum* (Table 4), such as 5,6-dihydroxy-4-methoxy-2H-pyran-2-one **(5)**, which is classified as a mycotoxin according to the Human Metabolome Database^1^ (HMDB) (Faid and Tantaoui-Elaraki, 1989), low amounts of deoxybrevianamide E **(1)** and dehydrodeoxybrevianamide E **(2)**, common metabolites from *Aspergillus ustus* (Scott et al., 1974), and 4-methoxy-6-n-propenyl-2-pyrone **(6)** as well as new compounds such as PI-1 **(7)** PI-2 **(8)**, PI-3 **(9)**, and PI-4 **(10)** (Arai et al., 1989). Whilst searching for NPs produced by species of *Penicillium*, Frisvad and Samson (2004) listed the NPs already mentioned as well as other NPs, such as arabenoic acid **(12)**, dehydrofulvic acid **(11)**, formylxanthocillin X **(4)**, 5-hydroxymethylfuric acid **(13)**, and other metabolites, which could not be identified. In this study, Frisvad et al. (2004) pointed out NPs that could be characterized as mycotoxins **(5, 6**, and **7)**, herbicides **(1, 12**, and **11)** and those with potential antibiotic activity **(2, 6**, and **13)**. Although these metabolites have been detected *in vitro* in artificial culture media, these metabolites have not been linked to infection yet and many other metabolites remain to be identified (Frisvad et al., 2004). In addition, there are no studies concerning the toxicity of these NPs against humans, plants, animals and bacteria (Faid and Tantaoui-Elaraki, 1989).

**Table 4:** Structure of the secondary metabolites produced by *P. italicum* already identified.

**N°**	**Secondary metabolite**	**Chemical structure**	**References**
**1**	Deoxybrevianamide E	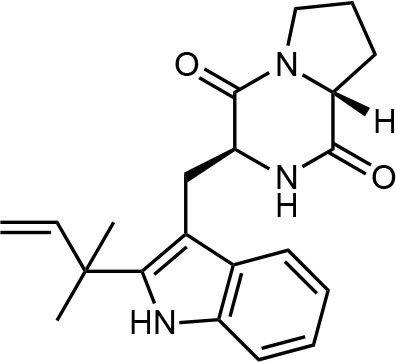	Scott et al., 1974; Arai et al., 1989; Frisvad et al., 2004; Smedsgaard et al., 2004
**2**	Dehydrodeoxybrevianamide E	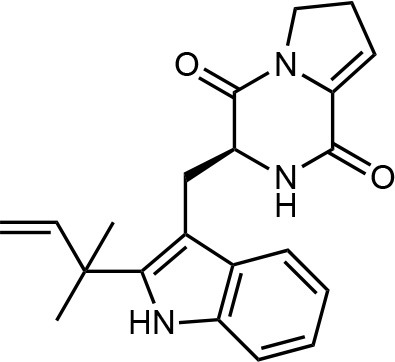	Scott et al., 1974
**3**	Xanthocyllin X	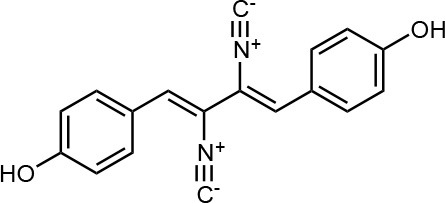	Frisvad et al., 2004
**4**	Formylxanthocillin X	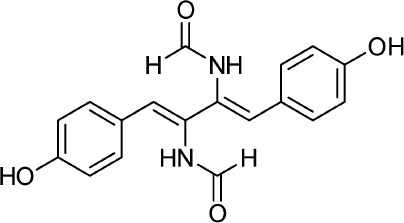	Frisvad et al., 2004
**5**	5,6-dihydroxy-4- methoxy-2H-pyran-2-one	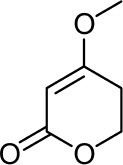	Faid and Tantaoui-Elaraki, 1989
**6**	4-methoxy-6-n-propenyl-2-pyrone	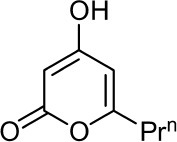	Arai et al., 1989
**7**	PI-1	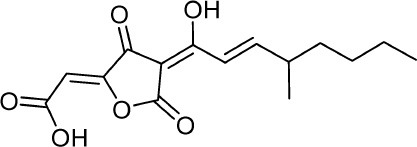	Arai et al., 1989
**8**	PI-2	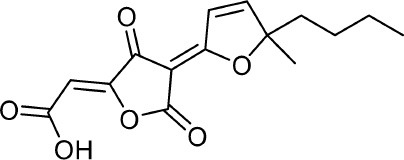	Arai et al., 1989
**9**	PI-3	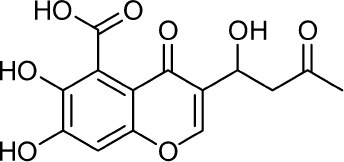	Arai et al., 1989; Frisvad et al., 2004
**10**	PI-4	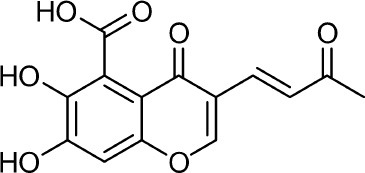	Arai et al., 1989
**11**	Dehydrofulvic acid	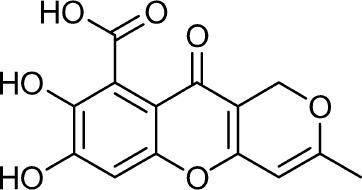	Frisvad et al., 2004
**12**	Arabenoic acid	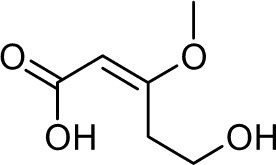	Frisvad et al., 2004
**13**	5-hydroxymethylfuric acid	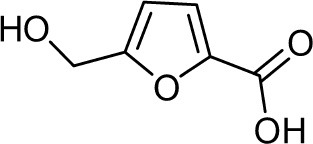	Frisvad et al., 2004

The *P. italicum* genome has already been sequenced, however, the biosynthetic potential encoded is still untapped. It is considered a necrotrophic plant pathogen and, due to the relationship between host variety and genome size for fruit pathogens, as proposed by studies such as Ballester et al. (2015) and Marcet-Houben et al. (2012), *P. italicum* has an intermediate host range (genome of about 29 Mb), meaning it can infect other fruits, despite having a larger pathogenicity in citrus fruits. *P. digitatum*, for example, has a smaller genome size (genome of about 25.7 Mb) being only capable of infecting citrus fruits (Ropars et al., 2016).

Li B. et al. (2015) sequenced the genome of three phytopathogenic fungal species of the genus *Penicillium* and functional analyses revealed interesting aspects about the biosynthesis of secondary metabolites and pathogenicity in the fungi *P. expansum* (apple pathogen), *P. digitatum*, and *P italicum*. Comparative genomic analysis of the three phytopathogens evaluated the natural products biosynthetic gene clusters (BGCs), using the antiSMASH bioinformatics program, revealing 55 BGCs in the genome of *P. expansum*, compared with 30 in *P. italicum*, and 24 clusters in *P. digitatum*. According to Li B. et al. (2015), some biosynthetic gene clusters, interestingly, are shared by all three or two of the studied *Penicillium* species, indicating that the species have chemically similar secondary metabolite production potential. Comparative analysis of the genome also indicated 10 clusters of biosynthetic genes shared by *P. digitatum* and *P. italicum*, pathogens that have great specificity with the citrus host. Such metabolites could be important for their virulence/pathogenicity and the biosynthetic studies of these compounds, as well as functional analyses, would be important, contributing to the understanding of the relationship between host-pathogen. The understanding of the active secondary metabolites involved in the pathogen-host interaction could increase the knowledge of the disease and, in the future, be used to research safe ways of control.

Studies in this field could contribute to a higher understanding of the fungal metabolomic profile. Comparative analyses of the DNA isolated from *P. italicum* were performed by Akhtar et al. (2013), managing to characterize the fungus from different locations and observe the mutations suffered through the RAPD (Random Amplified Polymorphic DNA) technique. The isolation of the CYP51 gene, which encodes eburicol 14-αdemethylase (P450_14DM_) in *P. italicum* was studied by Nistelrooy et al. (1996) in order to understand better the selective activity of demethylation inhibitors (DMIs), which were a promising antifungal not only for its selectivity but also because it is highly economical and meets the registration standards, as well as presenting already known fungal resistance mechanisms. DMIs were effective inhibitors of P450_14DM_ activity in *P. italicum* (Nistelrooy et al., 1996). However, it is now known that these fungicides are outdated, as previously discussed, being toxic to humans and the environment, in addition to the fact that *P. italicum* and other pathogens have already developed resistance to them.

## Conclusion

The fungus *P. italicum* is one of the main responsible pathogen for post-harvest diseases in oranges. It is responsible for significant drops in fruit production, and directly affects the economy of many countries, especially Brazil, as it is the largest producer and exporter of this product in the world. Since common control methods used today are quite toxic to human health, their application has been increasingly controlled, also considering that the fungus is developing a higher resistance to them. Alternative methods are being explored to replace them. The most promising ones are “killer” yeasts, such as *S. schoenii*; physical methods, such as UV and hot water treatment; essential oils, such as thymol and carvacrol; and volatile substances, such as dimethyl trisulfide. However, to this date, there are not enough studies on the effectiveness of these methods on a large-scale production or on their mechanisms of action against the fungus, containing possible undesirable effects, or even significant risks to human and plant health. For this reason, some studies have already been focused on understanding how *P. italicum* infects the fruit; its virulence factors (in which the PG enzyme seems to be the main responsible factor); the secondary metabolites produced, such as Italian acids, PI-1-4 and others mentioned in this article, which represent few NPs identified in comparison to the fungus cryptic biosynthetic potential, in addition to the lack of studies to prove which of them are directly linked to the infection; and the genomic aspect of the fungus, which has already clarified the selective activity of the DMIs and also *P. italicum's* potential for producing chemically similar NPs to other species of the *Penicillium* genus. Although the studies presented in this article have helped to understand better this phytopathological interaction, further investigations are still essential, since there are many aspects to be explored, especially concerning the fungus genome and their relationship with cryptic secondary metabolites. Understanding the biological role of these molecules in the pathogen-host interaction is essential for the development of new, more effective, nontoxic, and economically viable on large-scale control methods.

## Author Contributions

All authors listed have made a substantial, direct and intellectual contribution to the work, and approved it for publication.

## Conflict of Interest

The authors declare that the research was conducted in the absence of any commercial or financial relationships that could be construed as a potential conflict of interest.

## Footnotes

^1^Available online at: https://hmdb.ca/metabolites/HMDB0033517#ontology (accessed February 20, 2020).
